# Coenzyme Q10 in Mitochondrial and Lysosomal Disorders

**DOI:** 10.3390/jcm10091970

**Published:** 2021-05-04

**Authors:** Iain P. Hargreaves

**Affiliations:** School of Pharmacy and Biomolecular Science, Liverpool John Moore University, Byrom Street, Liverpool L3 3AF, UK; i.hargreaves@ucl.ac.uk

Within the mitochondrial respiratory chain (MRC), coenzyme Q10 (CoQ10) plays a key role as an electron carrier transporting electron derived from complex I (NADH: Ubiquinone reductase) and complex II (succinate: Ubiquinone oxidoreductase) to complex III (ubiquinol: Cytochrome c reductase). In addition, CoQ10 also serves as a potent lipid-soluble antioxidant protecting cellular membranes and circulatory lipoproteins against free radical induced oxidative damage [1]. The antioxidant function of CoQ10 is ascribed to its fully reduced ubiquinol form [1]. Therefore, a deficiency in CoQ10 status would be expected to contribute to disease pathophysiology by causing a failure in energy metabolism as well as compromising cellular antioxidant capacity. Indeed, a number of diseases have been associated with a CoQ10 deficiency including the heterogeneous MRC disorders [2]. A deficit in cellular CoQ10 status can be caused by either a primary or secondary deficiency in CoQ10 status [2]. The former results from mutations in genes involved with the CoQ10 biosynthetic pathway, the latter is thought to be caused by the secondary consequences of disease pathophysiology or increased degradation of the isoprene molecule, although this still remains to be elucidated [2]. A small subset of MRC disorders are caused by primary defects in the CoQ10 biosynthetic pathway. However, the majority of MRC disorders are associated with a CoQ10 deficiency of unknown cause (secondary CoQ10 deficiency) [2]. The therapeutic potential elicited by CoQ10 supplementation in the treatment of patients with MRC disorders may reflect the restoration of an underlying CoQ10 deficiency, although some patients with no evidence of a CoQ10 deficiency have also been reported to benefit from CoQ10 supplementation [2]. The clinical benefit elicited by CoQ10 in this latter group of patients may reflect its ability to restore electron flow in the MRC as well as replenish cellular antioxidant capacity [2]. Whilst CoQ10 supplementation appears to improve some peripheral abnormalities in patients with MRC disorders, neurological symptoms appear to be only partially ameliorated [2]. At present, the reasons for the refractory nature of the neurological symptoms associated with MRC dysfunction to CoQ10 supplementation remains to be elucidated, although they may include the limited transfer of CoQ10 across the blood–brain barrier (BBB) [2]. The proposed limited transfer of CoQ10 across the BBB is supported by a recent study by Wainwright et al. (2020) [3], which indicated that although supplemented CoQ10 may be able to cross the BBB, it then simply effluxes back from the brain to the blood, resulting in no actual net transfer of exogenous CoQ10 into the central nervous system.

In addition to the MRC, CoQ10 also serves as both an electron and proton carrier in the lysosomal respiratory chain (LRC) which, together with the V-ATPase pump, maintains the acidity of this organelle (pH 4.0–5.1) [4]. Therefore, in addition to decreasing mitochondrial function, a deficit in CoQ10 status has the potential to impair lysosomal acidification, which has now been demonstrated in a neuronal cell model of CoQ10 deficiency [5]. Although, it is uncertain as of yet whether this CoQ10 deficiency-induced de-acidification would be sufficient to impair lysosomal function. A deficit in CoQ10 status has been reported in the lysosomal storage disorder (LSD), mucopolysaccharidosis type III (MPS III) [4]. Whilst the effect of this CoQ10 deficiency has yet to be investigated on the disease pathophysiology of MPS III, the origin of this deficit in CoQ10 status is thought to result from either oxidative stress-induced catabolism or the sequestering of pyridoxal 5-phosphate, the active form of vitamin B6 which is required for CoQ10 biosynthesis by the glycosaminoglycans which accumulate in MPS III [4]. Further studies are required to investigate the true incidence of CoQ10 deficiencies in LSDs, although evidence of a CoQ10 deficiency has now been reported in Niemann Pick type c [4].

In view of the evidence of mitochondrial dysfunction associated with some LSDs [4], treatment strategies that target the mitochondria may prove to be of therapeutic benefit to patients with these disorders. The inclusion of CoQ10 amongst these mitochondrial targeted therapies has the added advantage of providing an antioxidant capacity to the treatment, which may be important in view of the reported susceptibility of the lysosome-to-oxidative stress-induced damage [4]. Although, considering the lack of consensus on appropriate treatments to enhance mitochondrial function, new non-pharmacological therapies such as fluorescent light energy (FLE) may also be appropriate strategies to investigate since FLE has recently been shown to enhance oxidative phosphorylation under in vitro conditions without associated evidence cellular morbidity [6].

In conclusion, further work is required to investigate the prevalence of a CoQ10 deficiency in LSDs as well as elucidating the causes and consequences of a deficit in CoQ10 status in disease pathophysiology. Furthermore, appropriate strategies are also required to maximize the therapeutic potential of CoQ10 as well as enhancing its ability to cross the BBB.
